# Factors Associated With HIV Disclosure and HIV-Related Stigma Among Adolescents Living With HIV in Southwestern Uganda

**DOI:** 10.3389/fpsyt.2020.00772

**Published:** 2020-07-31

**Authors:** Proscovia Nabunya, William Byansi, Ozge Sensoy Bahar, Mary McKay, Fred M. Ssewamala, Christopher Damulira

**Affiliations:** ^1^Brown School, Washington University in St. Louis, St Louis, MO, United States; ^2^International Center for Child Health and Development (ICHAD), Brown School, Washington University in St. Louis, St. Louis, MO, United States

**Keywords:** HIV-stigma, HIV disclosure, HIV-positive adolescents, social support, Uganda

## Abstract

HIV-related stigma has been documented as one of the greatest obstacles to reducing HIV spread, engagement in HIV treatment, and poor mental health functioning among people living with HIV (PLWH). Although disclosure is important for people to receive social support, the fear of stigma and discrimination prevents PLWH from disclosing their status. For children and adolescents growing up with HIV –with no opportunity for normal transition through adolescence due to stigma, it is important to identify additional family and community support systems, to improve their acceptance and health outcomes, including mental health functioning. This study examined family communication and social support factors associated with HIV disclosure and HIV-related stigma among children and adolescents living with HIV in Uganda. Baseline data from an NICHD-funded *Suubi+Adherence* study (N=702) were analyzed. Adolescents (10–16 years) were eligible to participate if they were: 1) HIV positive and knew their HIV status, 2) prescribed antiretroviral therapy, 3) lived within a family, not an institution, and 4) enrolled in one of the 39 health centers in the study area. Multiple regression analyses were conducted to determine family communication (frequency and level of comfort communicating with caregiver), social support (perceived child-caregiver support and social support from classmates, close friends, teachers, and caregivers), associated with HIV disclosure, disclosure comfort, and HIV internalized and anticipated stigma. Results show that level of comfort communicating with a caregiver was significantly associated with how often children discussed their HIV status with other people (*B* = 0.02, 95% CI = 0.00, 0.03, p = 0.04), and level of HIV disclosure comfort (*B* = 0.08, 95% CI = 0.04, 0.13, p < 0.01). In addition, support from within the school environment, i.e., from teachers and classmates, was uniquely associated with both HIV disclosure and HIV-related stigma. Findings point to schools as potential for implementing HIV stigma-reduction programs. In addition, programming aimed at improving HIV care and treatment outcomes for adolescents living with HIV should consider incorporating both family communication strengthening and HIV-stigma reduction strategies in their efforts, in order to improve HIV health-related outcomes, including overall mental health functioning of HIV positive adolescents.

## Introduction

An estimated 1.8 million children below the age of 15 years are living with HIV worldwide (1). Sub-Saharan Africa (SSA) is heavily burdened by HIV, with 85% of new infections among adolescents happening in the region (1). Within SSA, Uganda has an HIV prevalence of 7.5% among 15- to 49-year-olds (2). In addition, Uganda also reports unprecedented numbers of HIV-infected children, with close to 150,000 children (age, 0–14 years) living with HIV (3). While availability and access to free antiretroviral therapy (ART) has decreased child mortality (4), it has increased the likelihood that a large number of children living with HIV (CLWH) will transition into adulthood with HIV as a chronic, highly stigmatized illness (5, 6). Unfortunately, the HIV/AIDS-related stigma they experience results in a lower quality of life (6). Yet, stigma-reduction interventions targeting CLWH in SSA are almost nonexistent (7, 8).

HIV stigma is associated with public blame and moral condemnation for contracting the disease (9–11). Although HIV-related stigma has been declining in SSA since 2000, it remains high in several countries (1). In Uganda –one of the SSA countries that has implemented the stigma index survey, 25% of respondents indicated that they would avoid buying vegetables from a vendor living with HIV, suggesting that many people still lack basic HIV knowledge and showing the level of stigma associated with HIV/AIDS. In addition, people living with HIV (PLWH) report experiencing discrimination in health care settings, including being denied health services because of their HIV status and health care professionals disclosing their HIV status without their consent (1). Indeed, stigma has been documented as one of the greatest obstacles to slowing HIV spread, by perpetuating the culture of silence and fear, and preventing individuals from testing and seeking health care (12).

Stigma can be manifested internally due to perceived negative public attitude and self-blame. These feelings, in turn, predict psychological distress, including depression and post-traumatic stress disorders (13, 14), feelings of loneliness and social isolation (15–17), poor treatment and adherence (14, 18, 19), poor HIV-related physical health (20); and increase the risk of loss to follow up among CLWH (21). In addition, stigma can also be manifested externally through negative stereotypes (sexual promiscuity and deviant sexual behaviors), prejudice (fear, aversion, hatred), and discrimination, all of which create social barriers including access to healthcare (22).

Adolescence is a challenging period, associated with social, emotional, and cognitive changes (23, 24). Hence, children and adolescents need additional support, including emotional support and acceptance from family and community members. Yet, many CLWH cannot count on the “normal” transition to adolescence due to stigma where community and family members ostracize them for being HIV positive (25). Many of these children live with extended family members after losing their parents to HIV, where stigma is further perpetuated through rejection, verbal insults, avoidance, and ostracism due to unfounded fears of infection (26, 27). Because of such environment, CLWH may not develop strong attachment bonds with extended family and fail to develop a positive self-concept (25). This unsupportive social environment increases the risk for mental distress, including depression and trauma (4).

### Social Support, Disclosure, and HIV-Related Stigma

The relationship between social support and HIV-related stigma has been documented. Stigma is associated with low social support and deteriorating physical and mental health functioning (28, 29). In turn, low social support is associated with lower engagement levels in HIV treatment, resulting into poor HIV-related health outcomes, including poor mental health functioning (30). In addition, previous studies have demonstrated that the negative impact of stigma extends to the individuals’ social connections i.e. stigma limit PLWH’s ability to seek and engage in new supportive relationships, especially due to lack of disclosure (31). As such, while disclosure is important for PLWH to access and receive social support, the fear of discrimination prevents them from disclosing their status (32, 33). Indeed, higher levels of internalized stigma have been associated with low levels of disclosure and social interactions (27, 34–36); which in turn affects adherence to antiretroviral therapy (37, 38). Taken together, these findings suggest that social support and disclosure are critical for HIV care and treatment, as well as ensuring positive HIV-related health outcomes, including mental health functioning. However, despite the literature documenting the close relationship between social support, HIV disclosure, and stigma, very few studies have examined social support factors associated with HIV disclosure and stigma, especially among CLWH (39).

### Theoretical Framework

Social support serves to protect individuals from potential negative effects of life stressors (40). People who are socially integrated and who experience supportive relationships have better physical and mental health outcomes (41–43). However, while PLWH may have to disclose their HIV status to receive support, they must also perceive that social support exist before they make the decision to disclose. Indeed, PLWH are more likely to weigh the costs and benefits associated with disclosure (44). For example, while disclosure may mean that an individual may no longer have to struggle with concealing a secret, they may be exposed to stigma as a result (32, 33). Indeed, HIV nondisclosure is attributed to expectation of stigma (45). As such, PLWH with greater social support will have greater intention to disclosure their status (40, 46). On the other hand, individuals experiencing or anticipating stigma may be less likely to disclose their HIV status to others.

**Figure 1:**
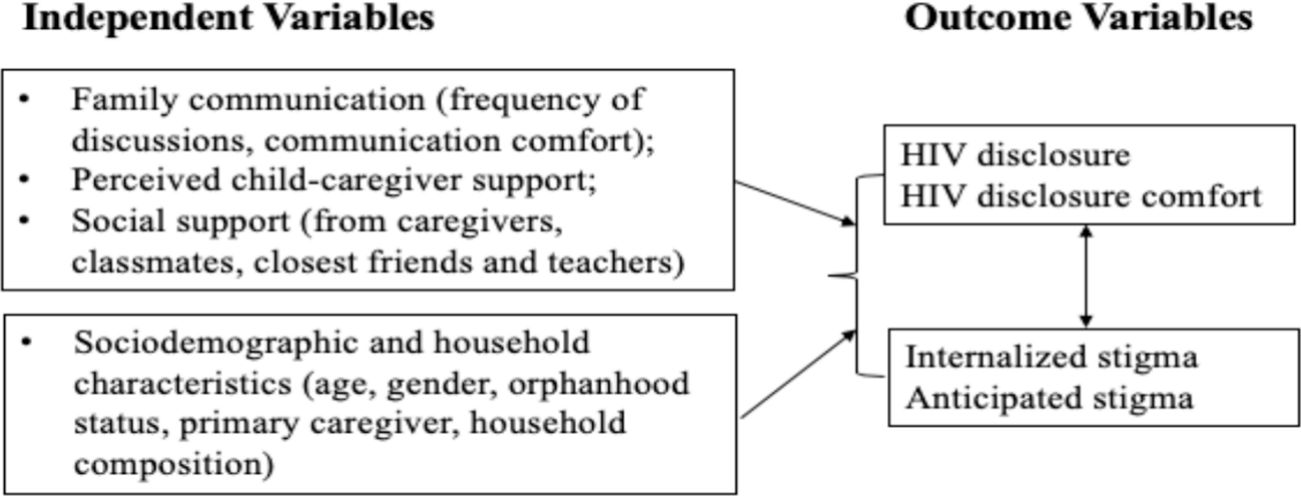
Conceptual Framework.

Guided by social support theory and based on previous studies discussed above, this study examines factors, including family communication and social support from multiple sources (i.e., caregivers, teachers, friends, and classmates) associated with HIV disclosure (keeping HIV a secret from others, and frequency of talking about HIV status) and HIV internalized and anticipated stigma. We expect that family communication and social support factors will be associated with higher levels of HIV disclosure and disclosure comfort, and low levels of HIV stigma. On the other hand, we expect that HIV stigma will be associated with low levels of HIV disclosure and disclosure comfort (**Figure 1**).

## Methodology

### Study Sample and Setting

This study utilized data from the *Suubi+Adherence* study (2012–2018), a randomized clinical trial funded by the National Institute for Child Health and Human Development (NICHD, grant R01HD074949). The Suubi+Adherence study examined an innovative family-based economic empowerment intervention on ART adherence among perinatally HIV-infected adolescents in southern Uganda, a region heavily affected by HIV/AIDS. Uganda has a national HIV prevalence rate of 7.5% among adults aged 15 to 49 years, with a higher prevalence rates of 12% in the southern region where the study was implemented (2). A total of 702 adolescents living with HIV (ages 10–16 years at study initiation) were enrolled in the study. Adolescents were eligible to participate if they were: 1) HIV positive and knew their status, 2) prescribed ART, 3) lived within a family, broadly defined and not an institution, and 4) enrolled in one of the 39 health centers or clinics in Rakai, Masaka, Lwengo, Lyantonde, Bukomasimbi, and Kalungu Districts in Uganda, where the study was implemented. Health clinics were randomized to either the treatment arm receiving an economic empowerment intervention or the control arm receiving usual care for adolescents living with HIV in the region. Randomization was conducted at the clinic level to avoid cross-arm contamination. All adolescents meeting the inclusion criteria at a particular clinic were invited to participate in the study and assigned to the same study condition. Adolescents who were not disclosed to and those who were not prescribed ART were excluded. Details on participant recruitment are provided in the study protocol (47).

Participation in the study was voluntary. Written informed consent and assent were obtained from caregivers and adolescents respectively. In November 2012, the study received Institutional Review Board (IRB) approvals from Columbia University (AAAK3852), the Uganda National Council for Science and Technology (UNCST, SS 2969), and Makerere University School of Public Health Higher Degrees, Research, and Ethics Committee (210).

### Data Collection

This study utilized baseline data collected *via* a 90-minute interviewer-administered assessment survey by trained Ugandan interviewers. All interviewers completed CITI certificate and Good Clinical Practice training. The interviewers were fluent in both English and Luganda—the local language widely spoken in the study area. All instruments were translated into Luganda by trained individuals fluent in both English and Luganda. Translated versions were then back translated into English to ensure accuracy and were certified by the Makerere University Institute for Languages in Uganda. All measures used in the Suubi+Adherence study were adapted and tested among children affected by HIV (48–52).

### Measures

The primary outcomes are: 1) HIV disclosure, 2) Level of comfort disclosing HIV status, and 3) HIV-related stigma.

*HIV disclosure* was measured using 2 indicators: 1) Do you keep your HIV status a secret from others such as, friends and other family members? (1, Never to 5, Always), with a higher score indicating non-disclosure; and 2) How often do you talk to people about your HIV status? (1, Never to 5, All of the time), with a higher score indicating higher frequency of disclosure.

*Level of comfort disclosing HIV status* to others was assessed using 4-items asking participants how comfortable they felt about letting others know of their HIV status, such as other children in the school, friends, other family members, and girl/boyfriends, on a 4-point scale (1, very uncomfortable; 4, very comfortable). A summary score was created (Cronbach’s alpha = 0.72) with a higher score indicating a higher comfort level of HIV status disclosure.

*HIV-related stigma* was assessed by 9-items adapted from the Berger Stigma Scale (53), measuring both internalized and anticipated stigma. Participants were asked to indicate the extent to which they agreed with the statements that HIV positive people have made about themselves (internalized stigma), and how HIV affects people (anticipated stigma), on a 4-point scale (1, strongly disagree; 4, strongly agree). Sample items include: “I feel guilty about having HIV” and “Having HIV affects whether people like you or not.” Items in the inverse direction were reverse coded to create a summary score (Cronbach’s = 0.74), with a higher score indicating higher levels of internalized and anticipated stigma.

*Social support* was measured by support from multiple sources, and perceived child-caregiver support. First, *s*ocial support from multiple sources was assessed using 24 items adapted from the Friendship Qualities Scale (54). The scale assesses the impressions of the quality of children’s friendships and relationships with their caregivers, classmates, closest friends and teachers, on a 5-point scale (1, never; 5, always). Summary scores were created, with higher scores indicating higher levels of social support from multiple sources (Cronbach’s alpha = 0.76). Each source of support (e.g. parent/guardian, teacher, classmate, friend) was analyzed separately in the analysis. Second, perceived child-caregiver support and family cohesion were both measured using items adapted from the Family Environment Scale (55) and the Family Assessment Measure (56). To measure *perceived child-caregiver support*, participants were asked to rate the adults they live with, on each of the 18-items (Cronbach’s alpha = 0.76), on a 5-point scale (1, never; 5, always). Summary scores were generated, with higher scores indicating higher levels of perceived child-caregiver support.

*Family communication* was measured in two ways: 1) frequency of discussions related to sensitive topics with caregiver, and 2) level of comfort discussing sensitive topics with caregiver. Frequency of discussions was assessed for 11-topics (Cronbach’s alpha = 0.74), including HIV/AIDS and other sexually transmitted infections, puberty, education and future plans, and risk-taking behaviors, on a 5-point scale (1, never; 5, always). Participants were then asked to state their level of comfort discussing the above topics (Cronbach’s alpha = 0.76) with their caregivers, on a 4-point scale (1, very uncomfortable; 4, very comfortable). Summary scores were created for both measures, with higher scores indicating higher frequency and comfort levels of communication between the child and caregiver.

A number of participants’ sociodemographic and household characteristics were included in the model, including age, gender, orphanhood status, primary caregiver, and household composition (number of people in the household and number of children in the household).

### Analysis Procedures

All analyses were conducted in SPSS version 25. Bivariate analyses (independent sample t-tests and chi-square tests) were conducted on key sociodemographic and household characteristics, social support factors, family communication, HIV disclosure and HIV-related stigma. These were compared and contrasted by gender. Multiple regression analyses were conducted to determine family communication and social support factors associated with HIV disclosure (keeping HIV a secret from others, and frequency of talking about HIV status), level of disclosure comfort, HIV internalized stigma and anticipated stigma, controlling for participants’ sociodemographic and household characteristics. All predictors were selected based on previous literature (i.e. factors associated with disclosure and HIV-related stigma). Statistical significance was set a prior at the 5% level.

## Results

### Family Communication, Social Support, HIV Disclosure, and HIV Stigma

Sample sociodemographic and household characteristics are summarized in **Table 1**. Participants reported moderate levels of family communication and social support from their classmates, friends, teachers, and caregivers (**Table 2**). We observe statistically significant gender differences. Specifically, compared to boys, girls were more likely to report higher levels of communication frequency with their caregivers on specific topics (25.73 vs. 23.63, p<0.01), higher comfort levels communicating with their caregivers (23.84 vs. 22.23, p<0.01), and higher levels of social support from multiple sources, including from caregiver (24.05 vs. 22.24, p<0.01), teacher (23.34 vs. 22.45, p=0.02), friend (21.82 vs. 20.30, p<0.01) and classmate (22.95 vs. 21.66, p<0.01).

**Table 1:** Baseline Characteristics of the Sample (N = 702).

Variable	Total Sample, % (N)	Boys, %(n)	Girls, %(n)	χ^2/^*t*-value	*p*-value
Gender	100(702)	43.6(306)	56.4(396)		
Age (Mean, SD) (min/max: 10–16)	12.42(1.98)	12.28(1.90)	12.53(2.03)	−1.92	0.06
*Orphanhood status*				0.27	0.63
Orphan child	65.0(456)	66.0(202)	64(254)		
Non-orphan	35.0(246)	34.0(104)	36(142)		
*Household Characteristics*					
Primary caregiver				0.12	0.94
Biological parent	47.1(330)	46.7(143)	47.3(187)		
Grandparents	29.4(206)	29.1(89)	29.6(26.7)		
Other relative (siblings, aunt, uncle, other)	23.5(165)	24.2(74)	23.0(91)		
Number of people in HH (Mean, SD) (min/max: 2–18)	5.74(2.56)	5.72(2.58)	5.77(2.54)	−0.25	0.80
Number of children in HH (Mean, SD) (min/max: 1–14)	2.35(1.92)	2.18(1.81)	2.47(2.0)	−1.98	0.05

**Table 2:** Family Communication, Social Support, HIV Disclosure and HIV-Related Stigma (N = 702).

Variable	Total Sample, N = 702 (Mean, SD)	Boys (n=306)Mean (SD)	Girls (n=396)Mean (SD)	χ^2/^*t*-value	*p*-value
*Family Communication*					
Frequency of communication with caregiver (min/max: 10–55)	24.81(7.9)	23.63 (7.7)	25.73 (8.1)	−3.49	<0.01
Level of comfort communicating with caregiver (min/max: 10–44)	23.14 (5.9)	22.23 (5.9)	23.84 (5.9)	−3.59	<0.01
Perceived child-caregiver support (min/max: 31–86)	58.98 (9.7)	60.65 (10.2)	61.64 (9.5)	−1.33	0.18
*Social Support from Multiple Sources*					
Caregiver (min/max: 6–30)	23.26 (5.02)	22.24 (4.94)	24.05 (4.94)	−4.81	<0.01
Teacher (min/max: 10–30)	22.95 (4.66)	22.45 (4.51)	23.34 (4.74)	−2.36	0.02
Friend (min/max: 8–30)	21.16 (4.71)	20.30 (4.62)	21.82 (4.68)	−4.28	<0.01
Classmate (min/max: 8–30)	22.38 (4.64)	21.66 (4.63)	22.95 (4.58)	−3.44	<0.01
*HIV Disclosure*					
Do you keep your HIV status a secret from others? (min/max: 1–5)	2.96 (1.69)	2.81 (1.65)	3.07 (1.73)	–2.01	0.05
How often do you talk to people about your HIV status? (min/max: 1–5)	1.94 (1.12)	2.05 (1.19)	1.86 (1.06)	2.15	0.03
Level of comfort talking about HIV-status with others (min/max: 3–16)	7.32 (3.12)	7.90 (3.43)	6.87 (2.79)	4.39	<0.01
*HIV-Related Stigma*					
Internalized (min/max: 6–23)	12.19 (3.79)	12.19 (3.84)	12.18 (3.75)	0.02	0.99
Anticipated (min/max: 3-12)	6.41(2.92)	6.49(3.03)	6.35(2.8)	0.62	0.54

Regarding HIV disclosure, compared to boys, girls were more likely to keep their HIV status a secret from others compared to boys (3.07 vs. 2.81, p=0.05), and less likely to feel comfortable discussing their HIV status with others (6.87, vs.7.90, p<0.01). In terms of HIV-stigma, participants reported moderate levels of both internalized stigma (M=12.19, SD=3.79) and anticipated stigma (M= 6.41, SD =2.92). No statistically significant differences were observed between boys and girls.

### Regressions on HIV Disclosure and HIV Stigma

Results from multiple regression analyses assessing factors associated with HIV disclosure are presented in **Table 3**. Family communication and social support factors were not associated with HIV disclosure (i.e. keeping HIV a secret from others). Both internalized stigma (*B* = 0.70, 95%CI = 0.03, 0.11, p<0.01) and anticipated stigma (*B* = −0.06; 95% CI = −0.12 to −0.01, p = 0.04) were associated with keeping HIV a secret from others. Level of comfort communicating with caregiver (*B*= 0.02, 95%CI = 0.00, 0.03, p=0.04), social support from a teacher (*B* = −0.03; 95% CI = −0.06 to −0.01, p = 0.02), and social support from a classmate (*B* **=**0.04, 95%CI = 0.02, 0.07, p<0.01), were all associated with how often children talked to other people about their HIV status. Similarly, internalized stigma (*B* **=** −0.03, 95% CI = −0.06 to −0.01, p=0.03), anticipating stigma (*B*=0.07, 95%CI= 0.03, 0.10, p<0.01), and being a female child (*B* = −0.25, 95% CI = −0.43 to −0.06, p=0.01) were associated with how often children talked to other people about their HIV status. Level of comfort communicating with a caregiver (*B*= 0.08, 95% CI =0.04, 0.13, p<0.01), internalized stigma (*B* = 0.11, 95% CI = −0.19, −0.03, p<0.01), and anticipated stigma (*B* = 0.14, 95% CI = 0.04, 0.25, p<0.01), were all associated with HIV disclosure comfort.

**Table 3:** Family Communication and Social Support Factors Associated with HIV Disclosure and Disclosure Comfort (N = 702).

Variable	Do you keep your HIV status a secret from others?			How often do you talk to people about your HIV status?			Level of comfort talking about HIV status with others		
	*B* (95% CI)	*β*	*p*-value	*B* (95% CI)	*β*	*p*-value	*B* (95% CI)	*β*	*p-value*
Female child	0.08 (−0.20, 0.36)	0.02	0.57	−0.25 (−0.43, −0.06)	−0.11	0.01	−1.22 (−1.73, −0.71)	−0.19	<0.01
Age	0.04 (−0.03, 0.12)	0.05	0.26	−0.02 (−0.07, 0.02)	−0.04	0.33	0.07 (−0.20, 0.07)	−0.04	0.32
Orphaned child	−0.14 (−0.46, 0.18)	−0.04	0.39	0.03 (−0.18, 0.24)	0.01	0.78	−0.02 (−0.60, 0.56)	−0.01	0.60
*Household Characteristics*									
Primary caregiver (biological parent)	0.12 (−0.42, 0.19)	−0.03	0.45	0.08 (−0.28, 0.12)	0.04	0.42	0.19 (−0.74, 0.37)	0.03	0.51
Number of people in HH	−0.07 (−0.16, 0.03)	−0.10	0.17	−0.03 (−0.09, 0.04)	−0.06	0.39	0.05 (−0.12, 0.23)	0.04	0.55
Number of children in HH	0.04 (−0.08, 0.17)	0.05	0.50	−0.01 (−0.09, 0.08)	−0.01	0.92	−0.17 (−0.40, 0.06)	−0.10	0.16
*Family Communication*									
Frequency of communication	0.15 (−0.01, 0.03)	0.07	0.11	0.01 (−0.00, 0.02)	0.07	0.11	0.03 (−0.01, 0.06)	0.07	0.10
Level of comfort communicating	0.01 (−0.02, 0.03)	0.02	0.66	0.02 (0.00, 0.03)	0.09	0.04	0.08 (0.04, 0.13)	0.16	<0.01
*Social Support (ref. caregiver)*									
Teacher	0.03 (−0.01, 0.06)	0.07	0.20	−0.03 (−0.06, −0.01)	−0.13	0.02	−0.02 (−0.09, 0.05)	0.04	0.51
Friend	0.02 (−0.01, 0.05)	0.06	0.24	0.01 (−0.02, 0.03)	0.02	0.73	−0.01 (−0.06, 0.06)	−0.01	0.98
Classmate	0.03 (−0.01, 0.07)	0.09	0.08	0.04 (0.02, 0.07)	0.17	<0.01	0.05 (−0.02, 0.12)	0.07	0.17
Perceived child-caregiver support	−0.01 (−0.02, 0.01)	−0.03	0.55	0.00 (−0.01, 0.01)	0.01	0.96	−0.01 (−0.04, 0.04)	−0.01	0.87
*HIV-Related Stigma*									
Internalized stigma	0.70 (0.03, 0.11)	0.15	<0.01	−0.03 (−0.06, −0.01)	−0.11	0.03	0.11 (−0.19, −0.03)	−0.13	<0.01
Anticipated stigma	−0.06(−0.12, −0.01)	−0.10	0.04	0.07 (0.03, 0.10)	0.17	<0.01	0.14 (0.04, 0.25)	0.05	<0.01
Constant	0.42 (−1.27, 2.11)		0.80	1.49 (0.39, 2.60)		0.01	6.51 (3.43, 9.60)		<0.01
F-value (df)	2.90		<0.01	3.26		<0.01	4.08(14)		<0.01

B = unstandardized coefficient, β = standardized estimate.

Regarding HIV stigma (**Table 4**), frequency of communication with caregiver (*B*= 0.05, 95% CI = 0.01, 0.09, p=0.02), social support from a friend (*B* = −0.07, 95% CI = −0.15, −0.01, p=0.05), social support from a classmate (*B* = −0.09, 95% CI = −0.17, −0.01, p=0.03), higher levels of perceived child-caregiver support (*B*= −0.05, 95% CI = −0.09, −0.01, p=0.01), and HIV nondisclosure (*B* = 0.20, 95% CI = 0.03, 0.38, p=0.02), were all associated with internalized stigma. In addition, social support from a classmate (*B*= −0.08, 96% CI = −0.14, −0.01, p =0.02), and having a biological parent as the primary caregiver (*B* = −0.63, 95% CI = −1.14, −0.12, p = 0.02), were associated with lower levels of anticipated stigma. Frequency of HIV status disclosure was also associated with anticipated stigma (*B* = 0.26, 95% CI = 0.05, 0.47, p = 0.02). Disclosure comfort was not associated with HIV stigma.

**Table 4:** Family Communication and Social Support Factors Associated with HIV Internalized and Anticipated Stigma (N = 702).

Variable	Internalized Stigma			Anticipated Stigma		
	*B (95% CI)*	*β*	*p-value*	*B* (95% CI)	*β*	*p*-value
Female child	−0.07 (−0.69, 0.54)	−0.01	0.82	−0.02 (−0.45, 0.50)	−0.01	0.92
Age	−0.08 (−0.24, 0.24)	−0.04	0.35	0.05 (−0.08, 0.17)	0.03	0.44
Orphaned child	−0.46 (−1.14, 0.23)	−0.06	0.19	−0.50 (−1.03, 0.03)	−0.09	0.06
*Household Characteristics*						
Primary caregiver (biological parent)	−0.40 (−1.06, 0.26)	−0.05	0.24	0.63 (−1.14, −0.12)	−0.11	0.02
Number of people in HH	−0.09 (−0.30, 0.11)	−0.06	0.37	−0.13 (−0.29, 0.03)	−0.11	0.12
Number of children in HH	0.10 (−0.18, 0.37)	0.05	0.48	0.06 (−0.16, 0.16)	0.04	0.60
*Family Communication*						
Frequency of communication	0.05(0.01, 0.09)	0.10	0.02	0.01 (−0.03, 0.04)	0.01	0.81
Level of comfort communicating	0.01 (−0.04, 0.07)	0.02	0.69	0.01 (−0.04, 0.04)	0.02	0.97
*Social Support from Multiple Sources (ref. caregiver)*						
Teacher	−0.01 (−0.09, 0.07)	−0.01	0.82	−0.04 (−0.10, 0.02)	−0.07	0.22
Friend	−0.07 (−0.15, −0.01)	−0.09	0.05	−0.02 (−0.07, 0.04)	−0.02	0.60
Classmate	−0.09 (−0.17, −0.01)	−0.11	0.03	−0.08 (−0.14, −0.01)	−0.12	0.02
Perceived child-caregiver support	−0.05 (−0.09, −0.01)	−0.11	0.01	−0.02 (−0.06, 0.01)	−0.07	0.12
*HIV Disclosure*						
Keeping HIV a secret from others	0.20(0.03, 0.38)	0.09	0.02	−0.01 (−0.15, 0.12)	−0.01	0.85
Frequency of HIV status disclosure	0.02 (−0.25, 0.29)	0.01	0.90	0.26 (0.05, 0.47)	0.10	0.02
Disclosure comfort	−0.07 (−0.17, 0.03)	−0.06	0.16	0.04 (−0.04, 0.11)	0.04	0.34
Constant	19.38 (16.02, 22.74)		<0.01	10.62 (8.03, 13.21)		<0.01
F-value(df)	3.27 (15)		<0.01	3.12 (15)		<0.01

B = unstandardized coefficient, β = standardized estimate.

## Discussion

This paper examined family communication and social support factors associated with HIV disclosure and HIV-related stigma among adolescents living with HIV in southwestern Uganda.

Our findings indicate the following. First, girls report higher levels of family communication and social support from multiple sources compared to boys. One explanation could be the socialization of girls versus boys in Uganda. Girls tend to be supported more because they are expected to take care of others in the family, including becoming home makers in the absence of an adult or a parent (57, 58). On the other hand, boys are trained to become responsible for the wellbeing of the household, including becoming breadwinners—from an early age (59). Overall, this finding is consistent with other studies that have documented gender differences in social support and social networks—reporting that women tend to have larger and more varied social networks with more friends and more social support compared to men (60–62).

Second, even with high levels of social support, girls in our study were less likely to discuss their HIV status with others and to feel comfortable disclosing their status, compared to boys. This finding is in line with previous studies in SSA that have documented lower disclosure levels among women—specifically, due to their economic and social vulnerability relative to men, fear of rejection, abandonment or partner violence (63–65). Although non-disclosure could serve as a protective factor against stigma, it may also have implications for access and utilization of HIV care and treatment among adolescent girls (66). Specifically, if other family members and close friends have no knowledge of the adolescent’s HIV status, they are less likely to provide appropriate ongoing care and support, including support with their medication adherence.

Third, one of the major barriers to positive and effective parent-child sexuality and HIV communication in SSA is communication style. A review of communication processes and barriers to sexuality communication in SSA demonstrate that parent-child discussions tend to be authoritarian and unidirectional, characterized by vague warnings rather than direct, open discussions, making discussions with children very uncomfortable and ineffective (67). Moreover, studies in Uganda have documented that among caregivers of CLWH, caregiver communication about HIV knowledge and medication is generally low (68, 69). Indeed, our findings indicate that what is important is not the frequency of communication, but rather the level of comfort communicating with the caregiver on sensitive topics, including HIV/AIDS. More specifically, higher level of comfort communicating with a caregiver was associated with how often children discuss their HIV status with other people, including other family members and friends, as well as level of comfort disclosing their status. As such, understanding how parents and caregivers convey sexuality and HIV-related knowledge to their children is important for the success of HIV prevention programming targeting adolescents, including those living with HIV.

Fourth, support from within the school environment, including from teachers and classmates was associated with HIV disclosure and HIV stigma, respectively. On one hand, support from teachers was associated with HIV non-disclosure. It could be that in an attempt to shield children from HIV-related stigma, teachers are likely to advise them not to talk to others about their status (17). On the other hand, support from classmates and friends was associated with low levels of internalized and anticipated stigma. It could be that once adolescents feel supported by their close friends and classmates, they are less likely to worry about being stigmatized. This finding has important implications for schools as potential for stigma-reduction programming targeting adolescents living with HIV in SSA.

Fifth, our findings are consistent with studies that have documented the inverse relationship between stigma and disclosure (70). Specifically, both internalized and anticipated stigma were associated with HIV non-disclosure and low levels of disclosure comfort. Similarly, HIV non-disclosure and frequency of status disclosure were both associated with high levels of internalized and anticipated stigma. Indeed, a study conducted in Uganda among people living with HIV found that HIV internalized stigma significantly reduced the likelihood of disclosure (35). Moreover, this association was amplified by the social distance—as PLWH were more likely to disclose to their close networks (such as family members and sexual partners) compared to distant individuals like public disclosures.

### Limitations

Findings presented in this study require careful interpretation in light of the following limitations. First, our study is based on participant self-reports which are prone to social desirability bias. However, given that there was no incentive for participants to overestimate or underestimate their reports, we assume that social desirability was minimal. Second, responses are based on self-reports, yet we know that having multiple sources of information is necessary for triangulation. Specifically, information from teachers, classmates, and parents/caregivers might provide a better picture of the levels of social support and HIV-related stigma experienced by adolescents living with HIV. Third, we utilized a sample of adolescents accessing ART from rural health clinics in our catchment area. Adolescents receiving their medication through other networks i.e. those who depend on their caregivers to get their prescriptions and those in urban areas were not included. As such, our findings may not be representative of all adolescents living with HIV in Uganda. Fourth, measures of stigma did not capture other mechanisms, such as enacted stigma—which involves experiences of discrimination, stereotyping, and/or prejudice that one is subject to due to their HIV positive status. This mechanism is important –with potentially significant implications for children’s HIV-related physical health and mental health functioning.

## Implications and Conclusions

Despite the limitations above, our study findings have significant implications for practice. Schools may be potential for interventions and programs that target stigma-reduction among adolescents living with HIV. Moreover, schools are important because they attract all students who would otherwise not be reached through community interventions. In our sample, 87% of participants were enrolled in school. Thus, schools are a key setting for addressing stigma among children living with HIV. Moreover, schools eliminate barriers that limit access to services, including stigma, cost of treatment and lack of transport to treatment facilities where counseling services are provided. In addition, programs to support and strengthen caregiver communication with their HIV positive children adolescents are urgently warranted. Overall, programming aimed at improving HIV care and treatment outcomes for children and adolescents should consider incorporating strategies that strengthen family communication, especially around HIV knowledge, treatment adherence, disclosure, stigma, and social support, in order to improve HIV health-related outcomes, including overall mental health functioning of HIV positive children and adolescents. In addition, schools may be potential for interventions and programs that target stigma-reduction among adolescents living with HIV in developing countries, especially those in SSA.

## Data Availability Statement

The datasets generated for this study are available on request to the corresponding author.

## Ethics Statement

Participation in the study was voluntary. Informed consent and assent were obtained from caregivers and adolescents respectively. The study received Institutional Review Board (IRB) approval from Columbia University (AAAK3852), the Uganda National Council for Science and Technology (UNCST, SS 2969), and Makerere University School of Public Health Higher Degrees, Research and Ethics Committee (210).

## Author Contributions

PN conceptualized the manuscript, performed statistical analyses and wrote the first draft of the manuscript. WB wrote sections of the manuscript. FS conceptualized, designed, and obtained funding for the study. CD organized the database. OS and MM reviewed the manuscript for intellectual content and made significant additions to the manuscript. All authors contributed to the article and approved the submitted version.

## Conflict of Interest

The authors declare that the research was conducted in the absence of any commercial or financial relationships that could be construed as a potential conflict of interest.
